# In this month’s Bulletin

**DOI:** 10.2471/BLT.25.000325

**Published:** 2025-03-01

In the editorial section, Matthew F Daley and Jason M Glanz (178) outline the implications of research published in this issue on hepatitis B vaccination for preterm infants. Jintana Jankhotkaew et al. (179) call for papers for an upcoming theme issue on health system responses to demographic changes.

Gary Humphreys (182–183) reports on the use of friendship benches as part of the national mental health response in Zimbabwe. Salim ‘Slim’ and Quarraisha Abdool Karim talk to Gary Humphreys (184–186) about their research on preventing HIV infection and transmission. 

## Australia

### Hepatitis B vaccination of preterm infants 

Hannah J Morgan et al. (187–193) assess risks for bronchopulmonary dysplasia.

## Bangladesh, Brazil, China, Colombia, Honduras, India, Jamaica, Malawi, Malaysia, Mexico, Mozambique, Peru, Philippines, South Africa, Thailand, United Republic of Tanzania, Zambia 

### Mortality surveillance and pandemics

Chalapati Rao et al. (213–222) highlight national efforts to ensure better data on cause-of-death. 

## Papua New Guinea

### Triage systems for emergency care

Rob Mitchell et al. (204–212) evaluate the use of a new triage tool.

## Global

### How much surgery is needed?

Sabrina Wimmer et al. (194–203) estimate the global burden of emergency and operative conditions.

### Reducing the environmental impact of menstrual hygiene products

Mandip Aujla et al. (223–225) describe alternatives to plastic and other toxic components.

### Improving the International Health Regulations’ joint external evaluations

Lawrence R Stanberry et al. (226–228) propose a fourth indicator on vaccine uptake.

